# M. Jobert’s Method of Treating Stricture

**Published:** 1860-12

**Authors:** 


*M. Jobert's Method of Treating Stricture.*—The *France Medicale* of the 11th ult. contains a clinical account of M. Jobert de Lamballe's practice, at the Hotel Dieu de Paris, in the treatment of stricture. The French surgeon's opinions are rather sweeping, as he condemns pretty well every mode of treatment save his own, which is described as follows: Take an emplastic bougie, and soften the extremity at the flame of a lamp; then incorporate more or less alum with it, according to the effect you wish to produce, and gently carry down the instrument to the seat of the stricture. There leave it a quarter of an hour. After two or three applications (the intervals are not mentioned), inflammation of the mucous membrane and discharge follow; the canal becomes disgorged by this transitory catarrh, and a bougie with an olive-shaped extremity passes easily. The case quoted in support is in nowise conclusive, and we must submit that the alum incorporation does not seem to us to present any advantages—rather the contrary. This practice of M. Jobert somewhat resembles the new mode of treating gleet in Paris, alluded to by our own correspondent; but practitioners will probably require good proof that these methods are really efficacious, and that they produce no mischief, before they have recourse to them.

